# Bone Marrow Environment in Metastatic Neuroblastoma

**DOI:** 10.3390/cancers13102467

**Published:** 2021-05-19

**Authors:** Chiara Brignole, Fabio Pastorino, Patrizia Perri, Loredana Amoroso, Veronica Bensa, Enzo Calarco, Mirco Ponzoni, Maria Valeria Corrias

**Affiliations:** 1 Laboratory of Experimental Therapies in Oncology, IRCCS Istituto Giannina Gaslini, 16147 Genova, Italy; chiarabrignole@gaslini.org (C.B.); fabiopastorino@gaslini.org (F.P.); patriziaperri@gaslini.org (P.P.); veronicabensa@gaslini.org (V.B.); enzocalarco@gaslini.org (E.C.); mircoponzoni@gaslini.org (M.P.); 2 Pediatric Oncology, IRCCS Istituto Giannina Gaslini, 16147 Genova, Italy; loredanaamoroso@gaslini.org

**Keywords:** neuroblastoma, bone marrow, metastases

## Abstract

**Simple Summary:**

In children with metastatic neuroblastoma (NB) the overall survival is still poor despite aggressive multimodal therapies. Relapse and progression, which are the major causes of death, usually occur at the most common metastatic site, the bone marrow (BM). Thus, understanding the complex interaction between the BM-infiltrating NB cells and the BM environment may help identify the mechanisms that allow survival of the metastatic cells. Moreover, new therapeutics targeting the microenvironment and/or the resistance to treatment can lead to improved survival for these children. The complexity of the BM environment and the existence of multiple mechanisms, all favoring the escape of metastatic NB cells from immune recognition, stress the need to test synergistic immune therapy approaches.

**Abstract:**

The study of the interactions occurring in the BM environment has been facilitated by the peculiar nature of metastatic NB. In fact: (i) metastases are present at diagnosis; (ii) metastases are confined in a very specific tissue, the BM, suggestive of a strong attraction and possibility of survival; (iii) differently from adult cancers, NB metastases are available because the diagnostic procedures require morphological examination of BM; (iv) NB metastatic cells express surface antigens that allow enrichment of NB metastatic cells by immune–magnetic separation; and (v) patients with localized disease represent an internal control to discriminate specific alterations occurring in the metastatic niche from generic alterations determined by the neoplastic growth at the primary site. Here, we first review the information regarding the features of BM-infiltrating NB cells. Then, we focus on the alterations found in the BM of children with metastatic NB as compared to healthy children and children with localized NB. Specifically, information regarding all the BM cell populations and their sub-sets will be first examined in the context of BM microenvironment in metastatic NB. In the last part, the information regarding the soluble factors will be presented.

## 1. Neuroblastoma

Neuroblastoma (NB) is the second most common solid childhood tumor [1,2,3,4]. Tumors arise in the sympathetic nervous system; about 45% of cases arise in the adrenal medulla and, in the remaining cases, in the para-spinal ganglia at any possible location [5], thus, NB cells secrete the sympathetic catecholamine epinephrine and norepinephrine and their metabolites.

NB clinical presentation is highly heterogeneous, ranging from localized disease, requiring only local control by chemotherapy and surgery, to metastatic disease, present in 50% of cases already at onset, requiring multimodal aggressive treatment modalities [1,2,3,4]. Today, staging is based on the presence of surgical risk factors [6] and established prognostic factors [7]. Infant and children with NB are classified into very low-, low-, intermediate- and high-risk groups depending on the age at diagnosis, with a benign effect of age below 18 months, presence/absence of metastasis (stage M/stage L, respectively), and presence of genomic aberrations at relevant loci, such as the amplification of the *MYCN* oncogene or the deletion of chromosome 11q [7]. The outcome of NB patients is highly variable, reflecting the heterogeneity of the clinical presentation. The 5-year overall survival (OS) ranges from 98–100% for stage L infants (<18 months of age at diagnosis) without *MYCN* amplification [8] to 50% for children with stage M disease [9].

In children with stage M NB, the tissues mostly involved are the bone marrow (BM) and the bone. As the majority of patients with stage M NB present metastases already at onset, the in vivo evaluation of factors influencing BM and/or bone invasion by NB cells is quite difficult to address. However, the peculiar nature of stage M NB has facilitated the studies of the complex interactions between the metastatic NB cells and the bone and BM resident cells, as compared with other cancer types. According to clinical practice, morphological examination of BM smears and of bone trephine biopsies are performed at diagnosis and during treatment for metastatic disease evaluation [10,11,12]. These diagnostic procedures make it possible to preserve biological material for additional studies on both NB and BM resident cells. Furthermore, specific characteristics of NB cells allow the performing of biological studies, even on small quantities of biological material. In fact, NB cells selectively express surface antigens, such as the disialoganglioside GD2 [13] and the B7-H3 co-stimulatory molecules [14], that allow their enrichment by immune–magnetic separation [15,16]. Last and more importantly, children with localized stage L NB represent an internal control to recognize the alterations occurring in the metastatic niche from those determined by the neoplastic growth at the primary site.

Relapse or progression at the metastatic site are the major causes of death for NB [17,18], thus, understanding the complex interaction between the BM-infiltrating NB cells and the bone and BM resident cells may help identify new therapeutic targets, possibly leading to improvement of survival of children with metastatic NB.

## 2. Physiological BM and Bone Environment

**Figure 1:**
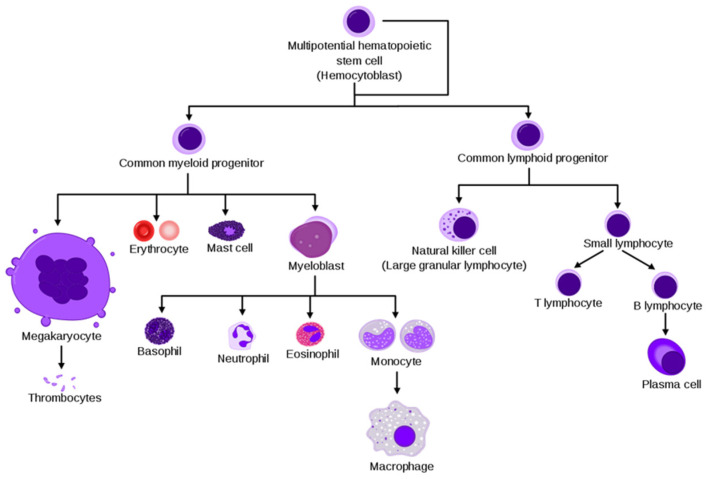
Simplified hematopoiesis hierarchy (Courtesy of Wikipedia).

BM is the primary site of hematopoiesis, where the self-renewal pluripotent hematopoietic stem cells (HSC) give rise, through committed precursors, to the erythroid (red cells and megakaryocytes), myeloid (granulocytes, monocytes, macrophages, and osteoclasts) and lymphoid (T, B and NK cells) lineages [19], as shown in Figure 1.

**Figure 2:**
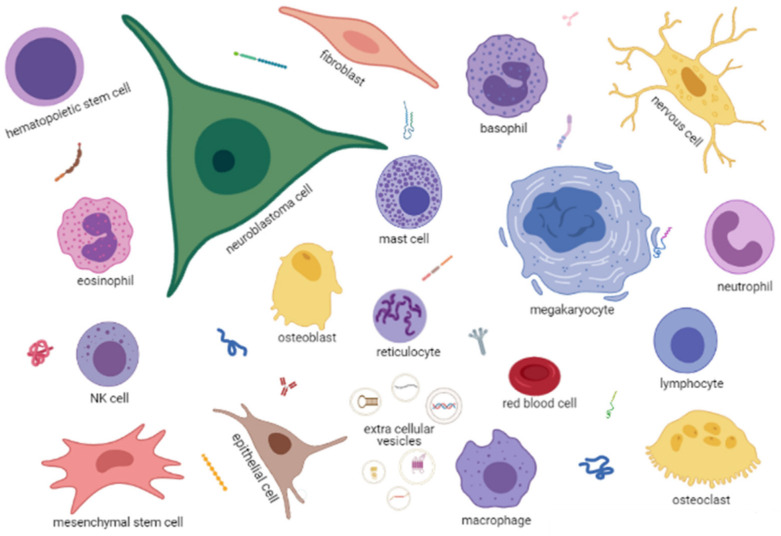
A cartoon, created in Biorender.com, depicting the BM microenvironment in NB. Each cell type is named in the figure. Extracellular vesicles of different dimensions with different types of cargo (DNA, miRNA, mRNA, proteins) are indicated in the bottom center of the figure. Different types of soluble proteins, such as antibodies, cytokines, enzymes etc., are scattered among the cells. Created with BioRender.com (accessed on 18 May 2021).

BM also contains non-hematopoietic cells, such as endothelial, nerve and mesenchymal stromal cells (MSC) that can differentiate in adipocytes, osteoblasts or chondroblasts (Figure 2). The BM is enclosed in bone structures that are continuously remodeled thanks to bone resorption mediated by the myeloid-derived osteoclasts and bone regeneration mediated by the MSC-derived osteoblasts [20].

Each cell type expresses receptors and ligands for other cells and may secrete soluble factors, such as growth factors, cytokines and chemokines, or extracellular vesicles of different sizes with various types of cargo, such as proteins, metabolites, DNA fragments or RNA species, including mRNA, microRNA or non-coding RNA (Figure 2).

BM homeostasis is, therefore, a process involving many cross-talks between hematopoietic and non-hematopoietic cells that are partially defined. The presence in this environment of a metastatic NB cell (Figure 2), expressing receptors/ligands, releasing soluble factors and extracellular vesicles, necessarily alters the physiological homeostasis, likely favoring its survival and proliferation.

Given the complexity of the BM microenvironment in NB, in this review, we first summarize the information regarding the features of the metastatic BM-infiltrating NB cells, with special attention to the mechanisms that allow them to escape immune recognition and to the differences with primary tumor cells that can be derived from the analysis of their gene expression profile.

Then, we summarize the alterations found in each of the BM cell populations in children with metastatic NB as compared to healthy children, whereas in the last part, we focus on the large variety of soluble factors, either free or enclosed in vesicles, that can modify the interaction between BM cells and the metastatic NB cells.

## 3. BM-Infiltrating Metastatic NB Cells

BM-infiltrating NB cells can be detected by flow cytometry of BM aspirates with the combined use of an antibody directed against antigens selectively expressed by NB cells, such as GD2 and B7-H3 [13,14], and an antibody directed against the differentiation cluster (CD) 45 expressed by all hematopoietic cells [21].

The percentage of CD45-/GD2+ BM-infiltrating NB cells is highly variable, ranging from 0.5–1% to 20–30% [15,16]. BM-infiltrating NB cells can also be detected in the cytospin of BM aspirates by immunocytochemistry with an anti-GD2 antibody [22] and by quantitative RT-PCR analysis for NB-specific mRNAs, such as tyrosine hydroxylase (TH), the first enzyme in the synthesis of catecholamine, and the paired-like homeobox 2b (PHOX2B), expressed at early stages during neuronal development [23]. Elevated levels of TH and PHOX2B mRNAs in BM aspirates from stage M NB children and infants at diagnosis are predictive of worse outcomes [24,25]. Similarly, elevated levels of a panel of 5 NB-specific mRNAs are predictive of worse progression-free survival in children with relapsed NB [26].

### 3.1. Mechanisms of Tumor Escape from Immune Recognition

BM-infiltrating NB cells, freshly isolated from BM, express co-stimulatory molecules, such as CD80, CD86, OX40L and 4-1BBL43 [27], but the absence of HLA class I molecules on the surface of NB cells, due to multifactorial mechanisms [28,29,30], does not allow their functional engagement by cytotoxic CD8+ T cells.

The absence of HLA class I molecules on the surface of NB cells makes them a target for NK cell cytotoxicity. NK cells, in fact, are inhibited by the engagement of inhibitory receptors of self HLA class I antigens [31]. BM-infiltrating NB cells, however, always express the co-stimulatory molecule B7-H3, a ligand for a still unknown inhibitory NK receptor that greatly reduces the killing activity of stimulated NK cells [32]. In addition, NB cells express little, if any, ligands for NK activating receptors. Precisely, most BM-infiltrating NB cells do not express PVR and DNAM molecules [33] ligand of the NKp30 and NKp44 receptors, nor the major histocompatibility complex class I-related chains, MIC-A and -B, ligands of the activating NKG2D receptor [34].

BM-infiltrating NB cells secrete the cytokine TGF-β that modifies, in a dose-dependent manner, the expression of the chemokine receptors CXCR4, CXCR3 and CX3CR1 in NK cells present in the BM of children with NB, potentially affecting their activity [35].

Collectively, these findings demonstrated that BM-infiltrating NB cells are endowed with several mechanisms to evade both T and NK cell immune surveillance.

### 3.2. Chemokine and Chemokine Receptors

Chemokines are a large family of small proteins that, upon binding to their cognate receptors, induce integrin activation and cytoskeletal rearrangement, promoting cell adhesion and directional migration [36].

BM-infiltrating NB cells express a broad range of chemokine receptors, including CCR1, CCR5, CCR6, CCR9, CXCR1, CXCR2, CXCR3, CXCR4, CXCR5 and CXCR6 [37]. However, most of these receptors seem to be involved in cell–cell adhesion in the BM, rather than in the NB cell migration towards the BM. In fact, no migration occurs when BM-infiltrating NB cells are exposed to CXCL16, the ligand of CXCR6 [37], and to CXCL12/SDF-1, the ligand of CXCR4 [38]. The only functional chemokine receptors expressed by BM-infiltrating NB cells are CXCR5, responsive in vitro to its ligand CXCL13and CXCR3, responsive to CXCL10 [37]. In the latter case, engagement of CXCR3 by CXCL10 mediates suppression, rather than enhancement, of NB cell proliferation [39].

### 3.3. Gene Expression Profiles

Starting from NB infiltrated BM samples, Hansford et al. obtained highly tumorigenic tumor spheres, suggesting that metastatic cells were enriched in tumor-initiating cells (TICs), endowed with the typical stem cell indefinite proliferative potential [40]. A gene expression profile of these cells was reported [41], but later, the isolated TICs were found not to be NB cells [42]. Moreover, it was demonstrated that in vitro expanded stem-like NB cells were a dynamic and heterogeneous cell population, quite difficult to characterize because of the influence of external stimuli [43].

Thus, to avoid any modification or selection following in vitro culture, we and others have decided to characterize freshly isolated BM-infiltrating NB cells using magnetic beads coated with anti-GD2 or anti-CD45 antibody (positive and negative immune selection, respectively).

GD2+/CD45− BM-infiltrating NB cells express the classical histological marker of NB, namely NB84 and CD56, and carry the same genetic alterations as the primary NB tumor cells [15,44]. The gene expression profile of freshly isolated BM-infiltrating NB cells has been compared to that of primary tumor NB cells [15] and to that of whole infiltrated BM samples [44]. In both cases, BM-infiltrating NB cells, also called disseminated tumor cells (DTCs), not surprisingly showed a lower expression of genes belonging to the angiogenesis and cell–cell adhesion functional annotation clusters. This finding was confirmed by the analysis of microRNA (miRNA) expression profiles of BM-infiltrating NB cells and primary tumor cells, demonstrating that the down-modulation of the focal adhesion pathway in the metastatic cells was regulated by CNOT1, a transcription regulator, through miR-659-3p [45].

Regarding the genes up-regulated in freshly isolated BM-infiltrating NB cells as compared to primary NB tumor cells, both studies found a higher expression of mitochondrial genes [15,44], suggesting a higher metabolic activity of the metastatic cells. Due to the different comparative analyses performed in the two studies, Morandi et al. were able to detect in BM-infiltrating NB cells the expression of genes typically expressed by resident BM cells [15]. To rule out the possibility that this finding was due to inefficient immune magnetic separation leading to contamination with resident BM cells, they performed a flow cytometry analysis of unprocessed BM aspirates, which confirmed that freshly isolated BM-infiltrating NB cells always express two molecules on their cell surface, namely the HLA-G molecule and calprotectin, an heterodimer encoded by the S100A8/A9 genes [15].

The expression of HLA-G and calprotectin by BM-infiltrating NB cells is intriguing. HLA-G is a tolerogenic HLA-class Ib molecule enabling the escape of tumor cells from immune surveillance [46]. Since HLA-G is not expressed by primary NB tumor cells [47], it can be postulated that the ability of BM-infiltrating NB cells to survive in the BM and restart proliferation after an apparent cure could be a consequence of the HLA-G immune suppressive activity.

Calprotectin is normally expressed by neutrophils and is released into biological fluids following their activation by inflammatory stimuli [48]. Calprotectin is a potent ligand of the Toll-like receptor 4 (TLR4), which is responsible for specific response to endogenous danger signals. The calprotectin-TLR4 axis was shown to drive metastatic invasion of lung cancer cells [49], but no information in NB is available. However, it can be hypothesized that calprotectin expressed by BM-infiltrating NB cells may be responsible in part for the state of chronic inflammation present in the BM environment in children with NB, as reported in detail in the cytokines’ paragraph.

### 3.4. Plasticity of NB Cells: Adrenergic and Mesenchymal Phenotypes

In the last few years, data are accumulating regarding the plasticity of NB cells and their ability to shift from an adrenergic to a mesenchymal phenotype and vice versa, under the control of core regulatory circuits regulating specific gene expression profiles [50,51,52,53,54,55]. Moreover, single-cell sequencing techniques, together with the availability of atlas describing the developmental fate of sympathetic progenitor cells [56], have allowed studies to better characterize the potential origin of neoplastic NB cells and to identify the mechanisms allowing the plasticity.

Presently, conflicting results have been published [57,58,59,60,61] regarding their developmental origin, but definitive results will certainly come in the next few years. As for the plasticity, it is interesting to note that already in 2007, Martinez et al. described the presence of GD2+/CD45− cells with mesenchymal phenotype in the BM of children with NB [62]. Whether these cells are indeed metastatic NB cells presenting with a mesenchymal phenotype will certainly be addressed in the near future.

### 3.5. Epithelial to Mesenchymal Transition (EMT)

EMT is a developmental process whereby stationary, adherent cells acquire the ability to migrate, thus, EMT can drive the metastatic process. In NB, EMT is associated with enhanced stem cell properties and drug resistance [63]. Recently, it has been shown in in vitro models of drug-resistant adrenergic (ADRN) and mesenchymal (MES) neuroblastoma cell lines that low expression of miR-124-3p is responsible for drug resistance and higher invasive capacity [64]. The development of organoids from patient-derived NB cells growing in an extracellular matrix of defined composition [65] will certainly help to elucidate the mechanisms that control BM invasion by NB cells.

## 4. BM Cellular Composition in NB

### 4.1. Lymphoid Lineage

BM-infiltrating NB cells do not seem to affect the lymphoid lineage in general [66,67], nor specific lymphocyte subsets, such as the immunosuppressive regulatory T cell, Treg and Tr1 [68]. No information on the features of BM NK cells is presently available, whereas an interesting lymphoid subset, the invariant NKT (iNKT) cells, has been extensively studied in NB tumors. iNKT cells express an invariant T cell receptor alfa chain that recognizes self or microbial glico-lipids presented by the monomorphic HLA-class I-like molecule CD1d [69]. Although NB cells do not express CD1d, iNKT cells exert their antitumor activity by killing the tumor-associated macrophages (TAM) [70]. The latter favors NB cell proliferation at the primary tumor site [71,72]. However, nothing is known regarding interactions among macrophages, iNKT cells and BM-infiltrating NB cells in the BM environment.

### 4.2. Myeloid Lineage

A particular cell of myeloid origin is represented by the osteoclasts that share with macrophages the CD115 receptor for the chemokine CCL2 [73]. NB cells suppress CCL2 expression that is necessary to promote infiltration by iNKT [74]. However, no specific information is available regarding these circuitries in the BM environment.

In NB, bone invasion by neoplastic cells seems to be predominantly osteolytic. NB cells express high levels of the receptor activator of nuclear factor-kB ligand (RANKL) that directly activates osteoclasts [75]. In addition, BM mesenchymal stem cells release several soluble osteoclast-activating factors, as detailed in the following paragraph. Bone resorption may create space for NB growth, but BM mesenchymal cells can differentiate in osteoblasts, giving rise to a vicious cycle of osteoblast and osteoclast genesis. One of the major players in this cycle is dickkopf 1 (Dkk1), an inhibitor of the canonical wingless (Wnt) pathway [76]. NB cells release Dkk1 [77], but its levels in children with NB do not associate with the presence of bone metastasis [78], making unlikely a pivotal role in NB bone invasion.

### 4.3. Erythroid Lineage

In children with NB, the maturation of erythrocytes was found to be selectively affected at the ortho-chromic stage, resulting in reduced erythrocyte count in the periphery [67]. Since children with localized NB share the same reduction, it is likely that primary tumor, rather than BM-infiltrating NB cells are responsible for this finding [67]. This hypothesis is supported by the fact that the major functional annotation clusters (anemia, blood group antigens, and heme and porphyrin biosynthesis) found under-expressed in the BM of children with NB, as compared to healthy children, were the same regardless of the presence/absence of metastatic BM-infiltrating NB cells [67,79].

### 4.4. Mesenchymal Stromal Cells

Among the non-hematopoietic cells, the MSCs are the most represented. MSCs completely lack the expression of hematopoietic markers, including CD45, and express CD105, CD73 and CD90.

BM MSCs have been shown to enhance NB growth in primary NB tumors and in vitro co-culture [80,81,82]. Only recently, the mesenchymal cells present in the BM environment have become the subject of specific studies. Hochheuser et al. found that the presence of BM-infiltrating NB cells is associated with an increased number of MSCs in the BM [66]. However, the fraction of MSCs expressing CD271, a marker of clonogenicity, was reduced and the MSC ability to differentiate was skewed towards osteoblasts [66]. Similar results were reported by Colletti et al. showing a higher osteogenic potential of MSC from BM infiltrated with NB cells, as compared to healthy BM MSC [83].

Hochheuser et al. also found a particular subset of BM MSC, negative for the clonogenic CD271 marker and positive for the osteogenic CD146 marker, associated with the presence of BM-infiltrating NB cells [66]. Interestingly, this subset was present at diagnosis and at relapse and disappeared during treatment. Most importantly, their presence after induction therapy is associated with an increased risk of relapse, suggesting a potential role in inducing chemoresistance [66]. Further studies are needed to clarify whether MSC favors BM infiltration by NB cells or NB cells alter the MSC differentiation process. Moreover, the use of mesenchymal cells as tumor-selective delivery vehicles for therapeutic compounds and oncolytic viruses needs to be reconsidered [84].

Very recent data presented at the Advances in Neuroblastoma Research 2021 by Burchill et al. showed the expression of PRRX1 and periostin mesenchymal markers in bone trephine biopsies and cytospins of BM aspirates from children with stage M NB [85]. Interestingly, elevated levels of BM infiltration with periostin-expressing cells is associated with a two-fold increased risk of an event, supporting a neoplastic potential for these MSC. Thus, whether the detected MSCs are true MSCs or NB cells with mesenchymal phenotype, as reported in the BM-infiltrating NB cell paragraph, needs to be addressed.

Finally, BM MSC can also affect response to anti-GD2 immunotherapy, nowadays included in the standard of care for children with stage M NB [86,87]. Wu et al. recently showed that the killing of NB cells by activated NK cells, through antibody-dependent cell-mediated cytotoxicity (ADCC) [88], can be suppressed by the presence of CD105-expressing BM MSC, and successfully restored by the use of an anti-CD105 antibody [89].

## 5. Soluble Factors

### 5.1. Catecholamines

Norepinephrine physiologically regulates HSC migration egress from the BM through anti-phase circadian fluctuations of Cxcl12 levels [90]. NB cells secrete norepinephrine, thus, Cxcl12 levels theoretically should be lower than in physiological conditions, potentially favoring hematopoietic cell egress. Conversely, no difference in the number of hematopoietic cells in the periphery was found [66,67], with the exception of erythrocytes [67], as reported in the erythroid lineage paragraph. These findings suggest that compensative pathways are in place in NB. In addition, we found that CXCL12 gene expression was low in all children with NB, regardless of the presence of BM-infiltrating cells [79]. Thus, the low Cxcl12 levels in the BM of NB patients seems to depend on the systemic effect of the primary tumor cells. The low Cxcl12 levels also make unlikely a pivotal role of the CXCL12/CXCR4 axis in promoting BM homing of the CXCR4 positive NB cells, as previously suggested [91]. Further supports for this conclusion are the finding, mentioned in the BM-infiltrating NB cell paragraph, that the CXCR4 receptor is not functional in those cells [38], and the results from Meier et al. that CXCR4 does not promote invasion [92].

### 5.2. Cytokines

Cytokines are proteins produced by various cell types that, following engagement of specific receptors, regulate the immune response and/or differentiation, proliferation and responsiveness of specific cell populations, mainly involved in immune responses [93]. 

Gene expression analysis of BM resident cells from children with NB demonstrated a significant over-expression of the interferon (IFN) and IFN-related DNA damage resistance (IRDR) signatures [79]. IFNs are pleiotropic cytokines involved in the innate immune responses against bacterial and viral pathogens. IFN-α and IFN-β (IFN type I), commonly induced by pathogens, are highly potent cytokines up-regulated during inflammation, whereas IFN-γ (IFN type II) is the master regulator of cytotoxic CD8+ T cell immune responses, including those against tumor cells [94]. Since the two signatures were also found in children with localized NB, it is conceivable that the primary tumor, rather than the BM-infiltrating NB, cells are responsible for the IFN-related gene up-regulation. It is interesting to note that activated NK cells secrete IFN-γ that, in turn, induces expression of HLA-class I [95] and PD-L1 [96] in freshly isolated BM-infiltrating NB cells. The up-regulation of HLA-class I would render the metastatic NB cells susceptible to cytotoxic CD8+ T cells, but the simultaneous up-regulation of PD-L1, the ligand for the immune check point PD-1, blocks the potential immune response.

NB cells secrete galectine-3 that, activating the galectine receptor on BM MSCs, induces the release of IL-6 [97,98]. IL-6 is responsible for the polarization of macrophages toward an M2 phenotype that, in turn, maintains a pro-inflammatory state with potential impairment of immune-recognition.

Other important immunosuppressive cytokines are IL-10, released by the regulatory T cells, and Arginase-1 (ARG-1) produced by immunosuppressive CD163+ macrophages. As compared to healthy children, the expression of ARG-1 gene in BM from children with NB was similar, whereas that of IL-10 gene was increased [99]. However, the Il-10 protein levels were not predictive of outcome, making its role in NB escape from immune recognition in the BM environment unlikely.

### 5.3. Monomorphic HLA Class Ib Molecules

A peculiar type of soluble factor endowed with tolerogenic properties is represented by the monomorphic HLA-class Ib molecules that display a limited number of alleles able to present antigens to cytotoxic CD8+ T cells [100]. The best characterized in NB are HLA-G, already described in the BM-infiltrating NB cell paragraph, and HLA-E and HLA-F. The soluble form of HLA-E and HLA-F seems to enhance immune recognition. Elevated plasma levels of both molecules, in fact, are significantly associated with better outcomes of children with NB [101], suggesting that these molecules were released by activated immune cells. BM plasma levels of HLA-G and HLA-E at diagnosis were detected in children with NB. Their levels were higher than in healthy children, but a potential association with outcome could not be assessed due to the design of the study [102].

### 5.4. Extracellular Vesicles

Extracellular vesicles (EVs) play an important role in cell–cell communications through spontaneous or receptor-mediated endocytosis, or by direct fusion with the plasma membrane [103]. EVs also represent an important delivery system in the tumor microenvironment, facilitating bi-directional signal dissemination between cancer and resident cells [104]. EVs include exosomes and microvesicles (MVs); exosomes are smaller than MVs (40–120 nm versus 50–1000 nm) and are generated through an endocytic pathway, whereas MVs are released from the plasma membrane [105]. In NB, MVs and exosomes have been isolated; both express the disialoganglioside GD2, confirming that they were released by the neoplastic NB cells [106,107]. EVs may be loaded with different types of cargo, as detailed below.

#### 5.4.1. Immunosuppressive Molecules

A soluble molecule endowed with immunosuppressive property is adenosine (ADO) generated by ectoenzymes that metabolize ATP. Two alternative pathways, one driven by CD39 and the other by CD38, converge to the activity of CD73 that converts AMP to ADO [108]. Recently, ADO and the various ectoenzymes involved in the pathways have been found in BM MVs from children with NB [109]. These vesicles strongly reduced the proliferation of T cells in vitro and were more abundant in the presence of BM-infiltrating NB cells. However, whether these vesicles were secreted by NB or by BM resident cells could not be determined [109].

#### 5.4.2. Proteins

The proteomic profile of exosomes derived by BM-infiltrating NB cells has been compared to that of exosomes generated by primary tumor NB cells [110]. Several proteins were found differentially expressed. Namely, exosomes isolated from BM-infiltrating NB cells contain more proteins involved in mitochondrial activity and fewer proteins involved in cell–cell adhesion and extracellular matrix assembly [110], fully confirming the results obtained by gene expression analysis of BM-infiltrating NB cells, in comparison with primary tumor cells [15,44].

#### 5.4.3. MicroRNA

MicroRNAs, important regulators of gene expression [111], have been found inside exosomes released by BM-infiltrating NB cells. Colletti et al. found elevated levels of miR-375 and demonstrated that this specific miRNA was responsible for the differentiation of BM MSCs in osteoblasts that may favor survival and proliferation of the metastatic cells [83].

Exosome-entrapped miRNAs were also found in blood from children with stage M NB [107]. The differential levels, before and after induction chemotherapy, of these exosome-entrapped miRNAs associate with response to therapy, suggesting a key role in inducing chemo-resistance [107].

Finally, miRNAs, known to be involved in mediating chemo-resistance of NB cells, have been searched in whole BM samples taken at diagnosis from children with stage M NB, but none was found differentially expressed in responders and non-responders to induction chemotherapy [112]. These latter results, however, were likely due to the fact that miRNAs produced by BM resident cells hid the variations occurring in miRNAs produced by the BM-infiltrating NB cells. Therefore, studies using purified BM extracellular vesicles are needed to better evaluate whether specific miRNAs can drive chemo-resistance of metastatic cells.

## 6. Conclusions

From the studies referenced here, several aspects of the BM microenvironment in metastatic neuroblastoma remain to be elucidated. In view of the importance of BM infiltration in the prognosis of children with NB, increased knowledge of the mechanisms involved in cell–cell interaction and on the role of soluble factors in mediating BM infiltration by NB cells and their survival and proliferation in BM, will allow finding new therapeutic targets and cure strategies for high-risk NB patients.

## Data Availability

Data availability must be asked to the referenced Authors.

## Abbreviations

ADCC	antibody-dependent cell-mediated cytotoxicity
ADO	adenosine
ARG1	arginase 1
B7-H3	B7 molecule homolog 3
BM	bone marrow
CCR	CC chemokine receptor
CD	cluster of differentiation
CSF-1	colony-stimulating factor–1
CXCR/L	CXC chemokine receptor/ligand
Dkk1	dickkopf 1
DNAM	DNAX accessory molecule
DTCs	disseminated tumor cells
EVs	extracellular vesicles
HLA	human leukocyte antigens
HSC	hematopoietic stem cells
IFN	interferon
IL	interleukin
iNKT	invariant NKT cells
IRDR	IFN-related damage resistance
MCP-1	monocyte chemoattractant protein-1
MIC-	major hystocompatibility complex class I chain related
miRNA	microRNA
MSCs	mesenchymal stromal cells
MVs	microvesicles
NB	neuroblastoma
NB84	neuroblastoma antigen 84
NK	natural killer cells
OS	overall survival
PD-1	programmed death- 1
PD-L1	programmed death-ligand 1
PHOX2B	paired-like homeobox 2b
PRRX1	paired related homeobox 1
PVR	poliovirus receptor
RANKL	receptor activator of nuclear factor-kB ligand
RT-PCR	reverse transcription-polymerase chain reaction
TAM	tumor-associated macrophages
TGF-β	tumor growth factor-β
TH	tyrosine hydroxylase
TICs	tumor-initiating cells
TLR4	toll-like receptor 4
Tr1	type 1 regulatory T cells
Treg	regulatory T cells
Wnt	wingless -int
